# Mr. Hastings on a Mode of Applying Blisters

**Published:** 1812-06

**Authors:** J. H. Hastings

**Affiliations:** 17, Haymarket


*To the Editors of the Medical and Physical Journal.*

GENTLEMEN,

BEING informed that the application of cantharides for the purpose of blistering, is frequently attended with effects (from the absorption of the flies producing much irritation) very unfavorable to the patient's case; and that to remove the objection would be very desirable; I beg permission, through the medium of your valuable Journal, to state, that I have found by covering the blistering part of the plaister with waxed paper, leaving the adhesive margin uncovered by it, and applying it to the skin with the waxed paper on, that a complete blister arises and clean, being free from the flies, and probably without any absorption of them.

It may be proper, perhaps, to state that the blister plaister should be very good, to answer the purpose.

The wax paper is prepared by putting demy paper on plates of copper or iron, over a fire, and rubbing the paper over with white wax inclosed in a piece of fine canvas, till the paper is fully charged with the wax.

I am, Gentlemen,

17, *Haymarket,* Your obedient Servant,

*April* 14*th.* J. H. HASTINGS.

